# Acute Polyradiculoneuropathy in a Patient With Squamous Cell Carcinoma of the Cervix: A Diagnostic Challenge

**DOI:** 10.7759/cureus.100832

**Published:** 2026-01-05

**Authors:** Imane Abourachida, Raymond Klevor, Mohamed Chraa, Najib Kissani, Nissrine Louhab

**Affiliations:** 1 Neurology, Mohammed VI University Medical Center, Marrakech, Marrakesh, MAR

**Keywords:** acute polyradiculoneuropathy, guillain-barré syndrome-like, gynecological cancers, paraneoplastic neurological syndromes, squamous cell carcinoma of the cervix

## Abstract

Acute polyradiculoneuropathies are a heterogeneous group of disorders that may present with rapidly progressive, symmetrical weakness and can mimic Guillain-Barré syndrome (GBS). We report the case of a 60-year-old woman who presented with acute ascending flaccid weakness of all four limbs, consistent with a severe acute polyradiculoneuropathy clinically resembling GBS. During the diagnostic workup, a previously unrecognized squamous cell carcinoma of the cervix was identified. This case report aims to describe a rare temporal association between an acute polyradiculoneuropathy with GBS-like features and cervical squamous cell carcinoma, while highlighting the diagnostic challenges and differential considerations in this clinical context. To our knowledge, this clinical association has been rarely reported in the literature.

## Introduction

Acute polyradiculoneuropathy (PRNA) is a neurologic emergency characterized by rapidly progressive limb weakness with hyporeflexia or areflexia, often accompanied by sensory symptoms and/or pain, reaching its nadir within days to four weeks. Diagnosis is primarily clinical and is supported by cerebrospinal fluid (CSF) findings (typically elevated protein with a normal or mildly increased cell count) and electrophysiological studies that help define demyelinating and axonal patterns [1,2].

Most PRNA presentations occur after a triggering event, most commonly an antecedent infection. Among reported infectious antecedents, *Campylobacter jejuni* is the most frequently identified bacterial trigger, described in a substantial proportion of patients across large series [3].

However, a clear infectious trigger is not always identified, and atypical contexts should prompt consideration of alternative immune-mediated drivers, including cancer-associated immune phenomena. Updated diagnostic frameworks for paraneoplastic neurologic syndromes integrate phenotype features, antibody risk category, and cancer association to estimate the likelihood of a paraneoplastic mechanism. In parallel, broader reviews of paraneoplastic disorders emphasize that peripheral nervous system involvement can occur and may complicate diagnostic attribution in oncologic settings [4]. Proposed mechanisms include immune cross-reactivity between tumor-associated antigens and peripheral nerve components, as discussed in contemporary overviews of immune-mediated neuropathies [5]. We report a patient with severe acute PRNA in whom subsequent workup revealed squamous cell carcinoma of the cervix, highlighting the diagnostic challenge of acute polyradiculoneuropathy occurring in an oncologic context and the importance of broadened etiologic evaluation when standard triggers are absent or the clinical setting is unusual.

## Case presentation

A 60-year-old woman presented to the neurological emergency department with a 5-day history of symmetrical, ascending weakness of the lower limbs. There were no associated sphincter disturbances or respiratory symptoms. Her medical history was notable for type 2 diabetes mellitus treated with oral antidiabetic agents and hypothyroidism treated with levothyroxine (25 µg/day). There was no relevant family history. She reported no recent respiratory or gastrointestinal infection and no recent vaccination. On examination, the patient was afebrile and in good general condition, with no dyspnea, lymphadenopathy, or skin abnormalities.

Neurological examination revealed symmetrical paraparesis, predominantly proximal, with a Medical Research Council (MRC) muscle strength grade of 3/5 in both lower limbs. Deep tendon reflexes were absent in the lower limbs and preserved and symmetrical in the upper limbs. Plantar responses were indifferent, and abdominal reflexes were present. Sensory examination was unremarkable on bedside testing. Cranial nerve examination was normal.

According to the electroneuromyography (ENMG) report, motor nerve conduction was slowed in all four limbs, associated with reduced compound muscle action potential amplitudes, prolonged distal motor latencies, and prolonged F-wave latencies. Sensory nerve conduction velocities were slowed, with reduced sensory nerve action potential amplitudes in all four limbs. No conduction block was observed. Overall, the ENMG report supported a diffuse sensorimotor neuropathic process; however, the absence of quantitative ENMG parameters and waveform tracings limited precise electrophysiological classification.

Cerebrospinal fluid (CSF) analysis showed no albuminocytologic dissociation, with a protein level of 0.2 g/L, a white blood cell count of 3 cells/mm³, and normal glucose levels. No abnormal cells were detected.

Although the clinical presentation raised suspicion for a Guillain-Barré-like syndrome, formal diagnostic criteria were not fully met, and alternative diagnoses were considered.

Laboratory investigations showed severe hypercalcemia with suppressed parathyroid hormone levels, associated with acute kidney injury and elevated inflammatory markers. Thyroid function tests showed a low free thyroxine level, with a normal thyroid-stimulating hormone (TSH). Cancer antigen 125 (CA-125) was mildly increased (61.08 U/mL). Other laboratory findings are summarized in Table 1.

**Table 1:** Laboratory findings at admission Laboratory findings at admission, including cerebrospinal fluid and serum analyses, with corresponding reference ranges. CSF: cerebrospinal fluid; GFR: glomerular filtration rate; CRP: C-reactive protein; ESR: erythrocyte sedimentation rate; PCT: procalcitonin; TSHus: ultrasensitive thyroid-stimulating hormone; CA125: cancer antigen 125; PTH-rp: parathyroid hormone–related peptide

Parameter	Patient value	Reference range
Proteinorachia	0.2 g/L	0.15–0.45 g/L
Cerebrospinal fluid (CSF) cytology	3 cells/mm³	0–5 cells/mm³
Cerebrospinal fluid (CSF) glucose	0.47 g/L	0.45–0.75 g/L
Total calcium	198.3 mg/L	85–105 mg/L
Phosphorus	56 mg/L	25–45 mg/L
Parathyroid hormone	7.9 pg/mL	15–65 pg/mL
Vitamin D (total)	< 8.10 ng/mL	30–50 ng/mL
Urea	76 mg/dL	7–20 mg/dL
Creatinine	14.2 mg/dL	0.6–1.3 mg/dL
Glomerular filtration rate (GFR)	54.11 mL/min/1.73 m²	> 90 mL/min/1.73 m²
24-hour proteinuria	0.16 g/24 h	< 0.15 g/24 h
24-hour diuresis	2000 mL	800–2000 mL
C-reactive protein (CRP)	47.1 mg/L	< 6 mg/L
Erythrocyte sedimentation rate (ESR)	78 mm/h	< 20 mm/h
Procalcitonin	4.55 ng/mL	< 0.5 ng/mL
TSHus	1.55 μIU/mL	0.4–4.5 μIU/mL
Free thyroxine (FT4)	0.58 μg/dL	0.8–1.8 μg/dL
Cortisol at 8 h	4.41 μg/dL	10–20 μg/dL
Cancer antigen 125 (CA-125)	61.08 U/mL	< 35 U/mL
PTHrP	18.48 pg/mL	< 20 pg/mL
Hemoglobin	9.2 g/dL	12–16 g/dL

**Figure 1:**
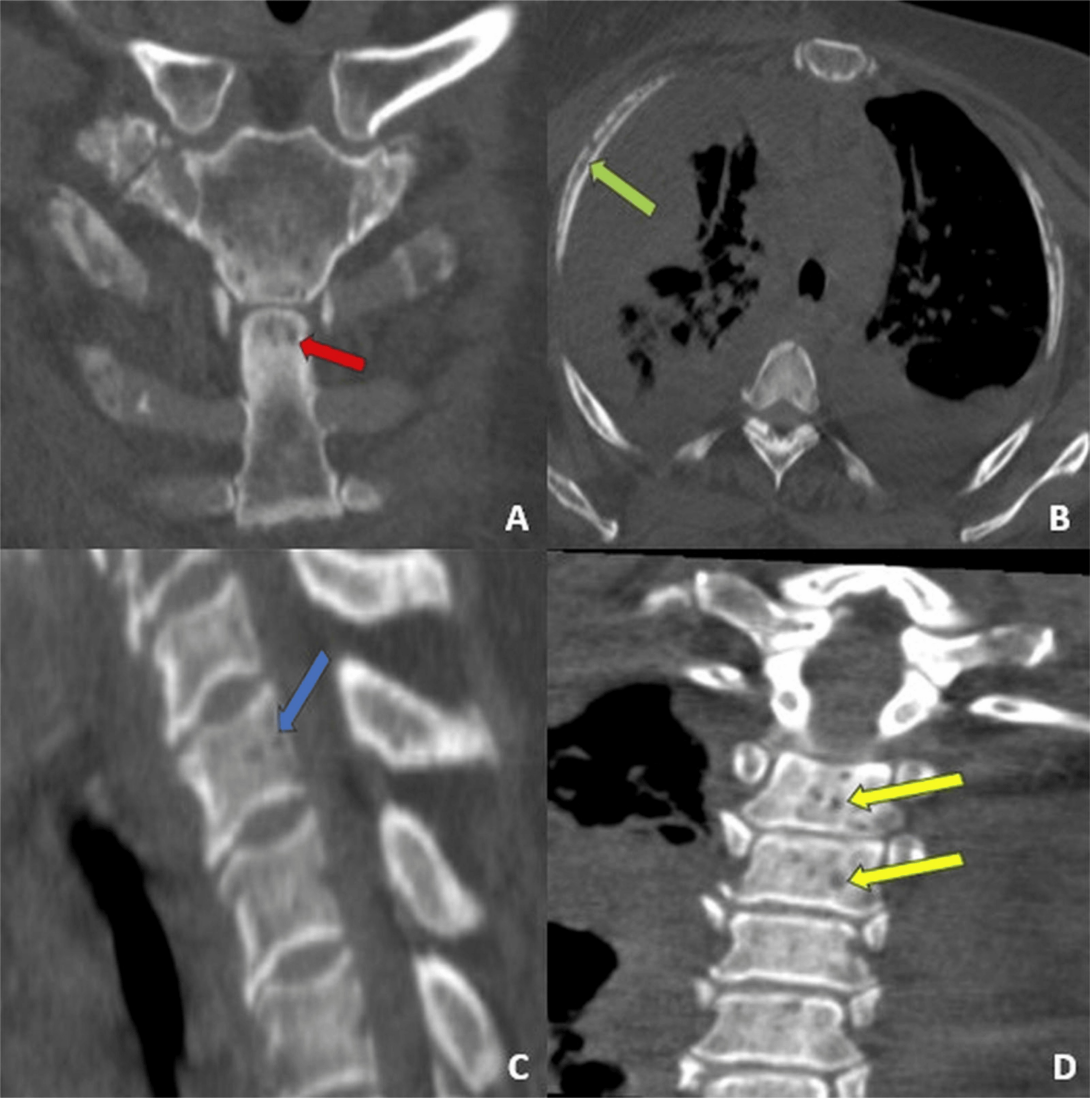
CT findings demonstrating multifocal osteolytic disease (A) Coronal CT image showing a lytic lesion of the sternum (red arrow); (B) Axial chest CT demonstrating an osteolytic lesion of the rib, suggestive of malignant involvement (green arrow); (C) Sagittal reconstruction revealing a vertebral osteolytic lesion with endplate erosion (blue arrow); (D) Coronal CT view highlighting multilevel vertebral body involvement with marked cortical disruption (yellow arrows)

Whole-body computed tomography (CT) revealed multiple osteolytic lesions involving the axial and appendicular skeleton (including the sternum, vertebral bodies, pubic symphysis, and iliac wings), some with cortical disruption. CT also showed a moderate volume of ascites with micronodular infiltration of the mesenteric fat, as well as moderate bilateral pleural effusions (Figure 1).

Renal ultrasound showed left hydronephrosis, with associated moderate fluid collection.

**Figure 2:**
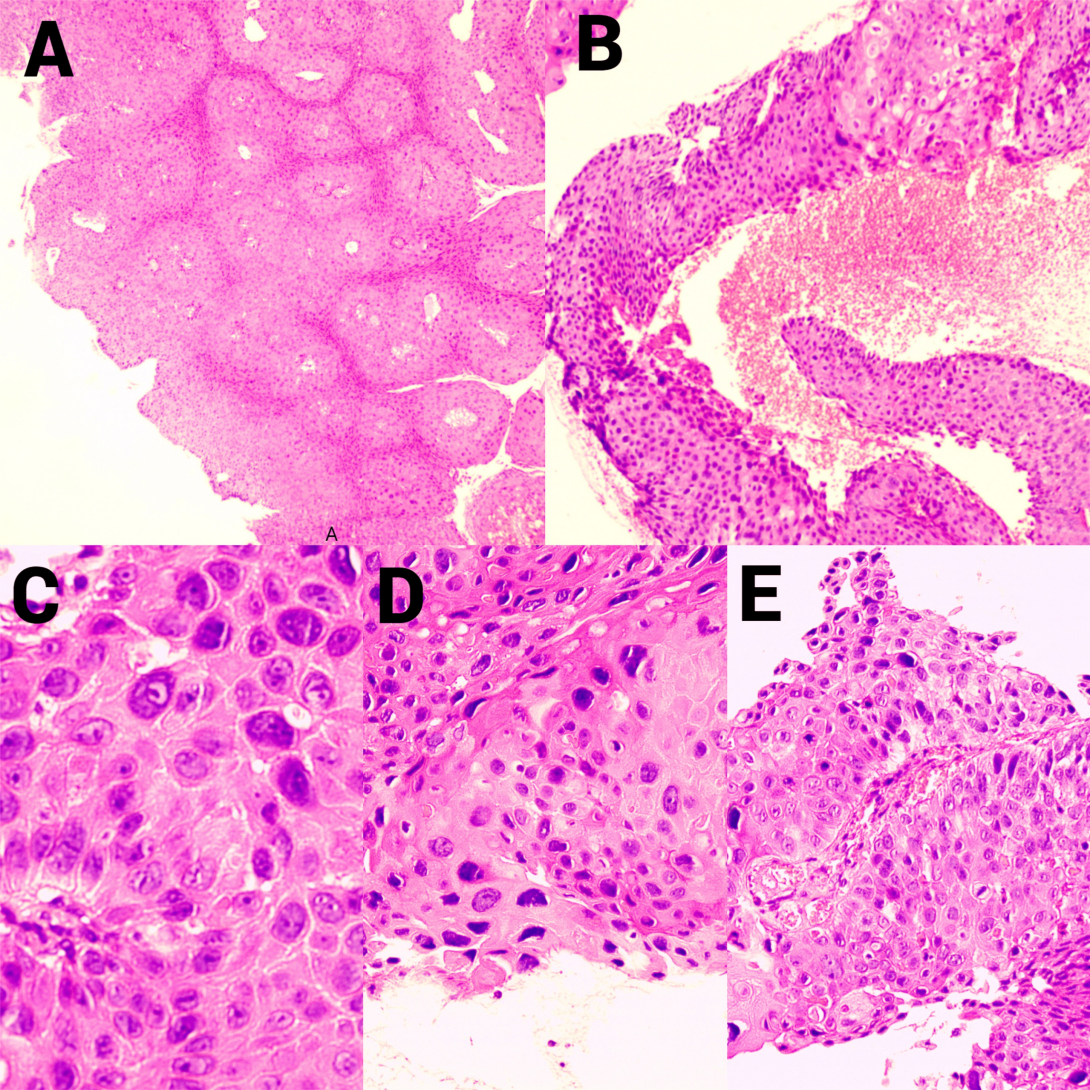
Histopathological features of cervical squamous cell carcinoma Carcinomatous proliferation of keratinizing squamous cells showing marked atypia and frequent mitoses (A) Low-power infiltrative nests (H&E ×4); (B) Focal keratinization (H&E ×10); (C) Pleomorphic tumor cells (H&E ×20); (D) Marked atypia (H&E ×40); (E) Dyskeratotic cells within cohesive nests (H&E ×20)

A cervical smear was suggestive of an infiltrating, moderately differentiated keratinizing squamous cell carcinoma. The diagnosis of cervical squamous cell carcinoma was subsequently confirmed on histopathological examination (Figure 2).

Hypercalcemia and acute kidney injury were managed during the initial hospital course with intravenous hydration and measures to treat hypercalcemia, with close monitoring of urine output and renal function. Hypercalcemia was corrected over the first three hospital days, without clear neurological improvement during that period. Inflammatory markers were elevated (CRP and procalcitonin), raising concern for infection; however, the patient remained afebrile with no clinical source identified and negative microbiological cultures. Given concomitant acute kidney injury and advanced malignancy, the specificity of procalcitonin was considered limited. Given the suspected acute polyradiculoneuropathy, intravenous immunoglobulin (IVIG) was initiated on hospital day 4 (approximately day 9 after symptom onset) at the standard regimen (0.4 g/kg/day for five days). Over the subsequent week, lower-limb strength improved clinically from MRC 3/5 to 4+/5. The patient’s hypothyroidism was chronic and treated with levothyroxine; thyroid function tests showed low free thyroxine with a normal TSH, a pattern unlikely to fully account for the acute presentation. The patient was transferred to oncology for staging and guideline-based systemic management of advanced cervical squamous cell carcinoma, along with supportive care. She later died from malignancy-related complications.

## Discussion

Acute polyradiculoneuropathy may occur in a wide range of systemic contexts, including malignancy. In rare reports, cancer-associated acute polyradiculoneuropathy has been hypothesized to involve immune-mediated mechanisms, potentially including immune cross-reactivity whereby antibodies directed against tumor-associated antigens may also recognize peripheral nerve components. Although paraneoplastic neurological syndromes (PNS) have been described in a variety of cancers, involvement of the peripheral nervous system remains exceptional [4,5]. Malignancies most frequently reported in association with acute immune-mediated neuropathies include lymphomas, leukemias, and epithelial tumors of the breast, lung, and colon [6].

The diagnosis of an acute polyradiculoneuropathy occurring in an oncologic context remains challenging and is primarily one of exclusion. Current diagnostic frameworks, including the 2021 updated criteria for PNS, emphasize the importance of a compatible neurological phenotype occurring in temporal association with cancer, with supportive evidence, such as tumor-related antibodies, when available [5,7]. However, the absence of identifiable onconeural antibodies does not preclude a paraneoplastic mechanism, particularly in peripheral neuropathies, where antibody associations are less well-defined. Importantly, no specific antibody profile has been consistently linked to GBS or GBS-like presentations.

In gynecologic malignancies, reported paraneoplastic antibodies, such as anti-Hu (ANNA-1), anti-Ri, anti-Yo, and anti-Ta, are more commonly associated with central nervous system involvement, including cerebellar degeneration, encephalitis, or sensory neuronopathy, rather than acute inflammatory polyradiculoneuropathy [8]. Paraneoplastic neurological complications appear to be particularly uncommon in cervical carcinoma compared with ovarian or breast cancer [9,10].

In the present case, the temporal association between neuropathy and newly discovered metastatic cervical carcinoma raises the possibility of a cancer-associated mechanism; however, multiple confounding factors prevent attributing a mechanistic causal link. Severe malignant hypercalcemia and acute renal failure were present at admission. Hypercalcemia is known to cause neuromuscular weakness, paresthesia, and altered consciousness, whereas renal failure may contribute to uremic neuropathy or encephalopathy. Both mechanisms, therefore, represent valid differential diagnoses and were integrated into the interpretation of the case. However, the presence of generalized areflexia and electrodiagnostic findings showing reduced amplitudes and slowing of conduction velocity, suggesting a sensorimotor polyneuropathy, supports involvement at the peripheral nerve or radicular level rather than a pure metabolic myopathy. Nonetheless, a conduction block was not detected, and cerebrospinal fluid analysis did not reveal albuminocytologic dissociation, which indicates that classical inflammatory demyelinating neuropathy criteria were not fulfilled.

Additional considerations include pre-existing type 2 diabetes and hypothyroidism, both of which may predispose to chronic polyneuropathy; however, neither condition typically produces an abrupt, rapidly progressive course over days. Furthermore, endocrine-related neuropathy tends to be distal and slowly progressive, contrasting with the bilateral ascending progression observed in this case. ICU-acquired weakness and critical illness polyneuropathy also constitute alternative explanations; however, in this case, weakness preceded intensive care admission, making these etiologies less likely.

Across published case reports of cancer-associated acute polyradiculoneuropathy with a GBS-like phenotype, several patterns emerge. First, the neurological presentation is often severe, characterized by rapidly progressive symmetrical weakness, areflexia, and, in many cases, bulbar or respiratory involvement. Second, IVIG remains the most commonly used treatment modality, either alone or in combination with tumor-directed therapy. Neurological outcomes vary widely, ranging from complete recovery to persistent disability or death, suggesting that prognosis depends not only on immunotherapy responsiveness but also on tumor aggressiveness and overall burden [11-19].

Notably, several cases occurred in the context of metastatic disease, emphasizing the need for clinicians to remain vigilant for paraneoplastic neuropathies in patients with advanced or immunologically active tumors. Although no specific antibody profile has been consistently associated with paraneoplastic PRNA, the clustering of cases around aggressive or immune-modulating malignancies suggests an immune-driven mechanism.

Taken together, this case illustrates an acute polyradiculoneuropathy occurring in temporal association with advanced cervical carcinoma. Rather than establishing a causal relationship, this report highlights the diagnostic complexity of acute neuropathies in oncologic settings and emphasizes the importance of considering underlying malignancy in elderly patients presenting with atypical acute polyneuropathies. Cautious interpretation and comprehensive evaluation remain essential to avoid overdiagnosis while ensuring appropriate multidisciplinary management.

## Conclusions

Our case illustrates an acute GBS-like polyradiculoneuropathy occurring in temporal association with squamous cell carcinoma of the cervix. This life-threatening presentation highlights the diagnostic complexity of acute neuropathies in oncologic settings and underscores the importance of early recognition, careful diagnostic evaluation, and multidisciplinary management.
